# Supplementary material for: Anomaly detection in virtual machine logs against irrelevant attribute interference
Source: PLoS One. 2025 Jan 7;20(1):e0315897. doi: 10.1371/journal.pone.0315897 (PMC11706483; doi:10.1371/journal.pone.0315897)
Supplement: S1 Appendix — (PDF) [file pone.0315897.s002.pdf]

Appendix of "Anomaly detection in virtual machine logs against irrelevant attribute interference"

Hao Zhang[1], Yun Zhou[2], Huahu Xu[1*], Jiangang Shi[3], Xinhua Lin[4], Yiqin Gao[4],

1 Shanghai University,China
2 Shanghai KingLong IoT Co., Ltd. , China
3 Shanghai Shangda Hairun Information System Co., Ltd., China
4 Shanghai Jiao Tong University, China

* huahuxu@shu.edu.cn

Appendix

This section contains Python code to run the two simulations that are used in this paper.

Sim 1: Data preprocessing

This example illustrates how we are doing data preprocessing. We convert the log data into numerical time series data, which serves as the input for LSTM and AE-based log data feature extraction algorithm.

```
import re
import pandas as pd
import os

def simplify_log(log_file):
    """
    Simplifies the log by replacing numbers with '*' and returns simplified log entries.

    Parameters:
        log_file (str): The path to the log file.

    Returns:
        list: Simplified log entries.
    """
    simplified_logs = []
    pattern = re.compile(r'\d{4}-\d{2}-\d{2}T\d{2}:\d{2}:\d{2}\.\d{3}Z\|\d+.*')

    with open(log_file, 'r') as file:
        for line in file:
            simplified_log = re.sub(r'\d+', '*', line)  # Replace numbers with '*'
            if simplified_log not in simplified_logs:  # Remove duplicates
                 simplified_logs.append(simplified_log)
    return simplified_logs
```

```python

def merge_clusters(clusters, T_sim):
    """
    Merges clusters based on similarity condition.

    Parameters:
        clusters (list): List of current clusters.
        T_sim (float): Similarity threshold for merging clusters.

    Returns:
        list: Merged clusters.
    """
    new_clusters = clusters.copy()
    merged = True

    while merged:
        merged = False
        for i in range(len(new_clusters)):
            for j in range(i + 1, len(new_clusters)):
                if similarity(new_clusters[i], new_clusters[j]) >= T_sim:
                    # Merge clusters
                    new_clusters.append(merge(new_clusters[i], new_clusters[j]))
                    del new_clusters[j]
                    del new_clusters[i]
                    merged = True
                    break
            if merged:
                break

    return new_clusters

def similarity(cluster1, cluster2):
    """
    Calculates the similarity between two clusters (dummy implementation).

    Parameters:
        cluster1: First cluster.
        cluster2: Second cluster.

    Returns:
        float: Similarity score.
    """
    # Placeholder for actual similarity logic
    return 0.8  # Assume a constant similarity for demonstration

def merge(cluster1, cluster2):
    """
    Merges two clusters into a new one.

    Parameters:
        cluster1: First cluster.
        cluster2: Second cluster.

    Returns:
```

```
        Merged cluster.
    """
    # Implement the merging logic (e.g., combine elements of both clusters)
    return cluster1 + cluster2  # Simple concatenation for demonstration

def batch_process_data():
    """
    Processes multiple log files from specified directories and simplifies them.

    Returns:
        list: A list of simplified log entries from all processed log files.
    """
    all_logs = []

    # List of directories and their respective log counts
    log_directories = {
        '50_199': 9,
        '51_106': 24,
        '51_107': 24,
        '124_25': 24,
        '164_4': 24,
        '164_6': 24,
        '170_30': 18,
        '171': 30,
        '171_57': 7,
        '171_67': 24,
        '172': 30,
        '173': 30,
        '174': 30,
        '175': 30,
        '176': 30,
        '176_9': 13,
        '177': 29,
        '178': 30,
        '179': 13
    }

    for folder, count in log_directories.items():
        print(folder)
        for i in range(count):
            log_path = f'logs/{folder}/{i}.log'  # Construct log file path
            logs = simplify_log(log_path)
            all_logs.extend(logs)  # Collect all simplified logs

    # Perform clustering on simplified logs (assuming logs can be treated as clusters)
    T_sim = 0.5  # Example threshold for similarity
    clusters = merge_clusters(all_logs, T_sim)

    return clusters


```

```python
def get_out_put_list(string_to_number, log_file):
    """
    Reads a log file and maps simplified log entries to corresponding numbers.

    Parameters:
        string_to_number (dict): A dictionary mapping simplified log strings to numbers.
        log_file (str): The path to the log file.

    Returns:
        list: A list of numbers corresponding to the simplified log entries.
    """
    output_list = []
    with open(log_file, 'r') as file:
        for line in file:
            simplified_log = re.sub(r'\d+', '*', line)  # Replace digits with '*'
            if simplified_log in string_to_number:
                output_list.append(string_to_number[simplified_log])
    return output_list

def create_string_to_number_mapping(diction):
    """
    Creates a mapping from simplified log strings to numbers.

    Parameters:
        diction (DataFrame): DataFrame containing the simplified logs.

    Returns:
        dict: A dictionary mapping simplified log strings to their indices.
    """
    return {row['logtempt']: index + 1 for index, row in diction.iterrows()}

def process_logs(directory, count, string_to_number):
    """
    Processes a set of log files in the specified directory.

    Parameters:
        directory (str): The directory containing the log files.
        count (int): The number of log files to process.
        string_to_number (dict): A mapping of simplified log strings to numbers.
    """
    for i in range(count):
        log_file = f'{directory}/{i}.log'  # Construct log file path
        out_list = get_out_put_list(string_to_number, log_file)
        output_df = pd.DataFrame({'Number': out_list})
        output_df.to_csv(f'{directory}/{i}.csv', index=False)

def batch_process_data_to_num():
```

```
    """
    Main function to process multiple sets of log files and generate output CSV files.
    """
    diction = pd.read_csv('dictionary.csv')  # Load the dictionary from CSV
    string_to_number = create_string_to_number_mapping(diction)  # Create mapping

    log_directories = {
        'logs/50_199': 9,
        'logs/51_106': 24,
        'logs/51_107': 24,
        'logs/124_25': 24,
        'logs/164_4': 24,
        'logs/164_6': 24,
        'logs/170_30': 18,
        'logs/171': 30,
        'logs/171_57': 7,
        'logs/171_67': 24,
        'logs/172': 30,
        'logs/173': 30,
        'logs/174': 30,
        'logs/175': 30,
        'logs/176': 30,
        'logs/176_9': 13,
        'logs/177': 29,
        'logs/178': 30,
        'logs/179': 13,
    }

    for directory, count in log_directories.items():
        print(f"Processing {directory.split('/')[-1]}")  # Print directory name
        process_logs(directory, count, string_to_number)  # Process each log directory


if __name__ == "__main__":
    final_logs = batch_process_data()
    sorted_logs = sorted(final_logs, key=len)

    # Create a DataFrame and save to CSV
    df = pd.DataFrame({'logtempt': sorted_logs})
    df.to_csv('dictionarycsv', index=False)
    print(df)
    batch_process_data_to_num()
```

Sim 2: LSTM and AE-based log data feature extraction

This example shows the feature extraction. LSTM-based AE network is used to extract the feature vectors of log data, which are then classified using an SVM classifier.

```
import pandas as pd
```

```python
from sklearn.preprocessing import StandardScaler
import numpy as np
from sklearn.svm import OneClassSVM
from tensorflow.keras.models import Sequential
from tensorflow.keras.layers import LSTM, RepeatVector, TimeDistributed
from keras.layers import Dense

def get_train():
    for i in range(28, 411):
        csvfile = 'logs/Training_csv/' + str(i) + '.csv'  # Log location
        df = pd.read_csv(csvfile)
        if i == 28:
            resultnp = convert_to_serial(df)
        else:
            resultnp = np.concatenate((resultnp, convert_to_serial(df)), axis=0)
    print(resultnp.shape)
    return resultnp

def get_test():
    for i in range(27):  # Adjusted to match the provided range
        csvfile = 'logs/Testing_csv/' + str(i) + '.csv'  # Log location
        df = pd.read_csv(csvfile)
        if i == 0:
            resultnp = convert_to_serial(df)
        else:
            resultnp = np.concatenate((resultnp, convert_to_serial(df)), axis=0)
    print(resultnp.shape)
    return resultnp

def convert_to_serial(dataframeobj):
    scaler = StandardScaler()
    new_data_frame = dataframeobj['Number'].to_numpy()
    new_data_frame = new_data_frame.reshape(-1, 1)
    data_scaled = scaler.fit_transform(new_data_frame)

    window_size = 10
    timesteps = 5

    X = []
    for i in range(len(data_scaled) - window_size - timesteps + 1):
        X.append(data_scaled[i:i+window_size])
    X = np.array(X)
    print(X.shape)
    return X

def build_lstm_autoencoder(window_size):
    model = Sequential()
    model.add(LSTM(64, activation='relu', input_shape=(window_size, 1), return_sequences=True))
    model.add(LSTM(32, activation='relu', return_sequences=False))
    model.add(RepeatVector(window_size))
```

```python
    model.add(LSTM(32, activation='relu', return_sequences=True))
    model.add(LSTM(64, activation='relu', return_sequences=True))
    model.add(TimeDistributed(Dense(1)))
    model.compile(optimizer='adam', loss='mse')
    return model

def train_autoencoder_model(model, train_x, epochs=10, batch_size=64):
    model.fit(train_x, train_x, epochs=epochs, batch_size=batch_size)
    return Sequential(model.layers[:3])  # Encoder model

def train_svm_on_latent_representation(encoder, train_data):
    latent_representation = encoder.predict(train_data)
    data_reshaped = latent_representation.reshape(latent_representation.shape[0], -1)

    svm_model = OneClassSVM(kernel='rbf', nu=0.0000001)
    svm_model.fit(data_reshaped)
    return svm_model

def predict_and_evaluate(encoder, svm_model, test_data):
    new_latent_representation = encoder.predict(test_data)
    new_data_reshaped = new_latent_representation.reshape(new_latent_representation.shape[0], -1)
    predictions = svm_model.predict(new_data_reshaped)
    return predictions

def main():
    window_size = 10

    print("Preparing training data...")
    train_x = get_train()

    print("Building and training LSTM autoencoder...")
    autoencoder_model = build_lstm_autoencoder(window_size)
    encoder = train_autoencoder_model(autoencoder_model, train_x)

    print("Training SVM on latent representations...")
    svm_model = train_svm_on_latent_representation(encoder, train_x)

    print("Preparing test data...")
    test_x = get_test()

    print("Evaluating model on test data...")
    predictions = predict_and_evaluate(encoder, svm_model, test_x)

    print("train_x shape is:", train_x.shape)
    print("test_x shape is:", test_x.shape)
    print("Predictions:", predictions)

# Run the main function
if __name__ == "__main__":
    main()
```
